# Single-round infectious rotaviruses with deletions of VP7 or VP4 genes, based on SA11 and WC3 strain backbones, and their potential use as viral vectors

**DOI:** 10.1371/journal.ppat.1013484

**Published:** 2025-09-15

**Authors:** Tomohiro Kotaki, Yuta Kanai, Megumi Onishi, Yusuke Sakai, Daisuke Motooka, Zelin Chen, Yasutaka Enoki, Sayuri Komatsu, Katsuhisa Hirai, Shohei Minami, Takahiro Kawagishi, Hiroshi Ushijima, Takeshi Kobayashi

**Affiliations:** 1 Department of Virology, Research Institute for Microbial Diseases, The University of Osaka, Osaka, Japan; 2 Department of Infectious Disease Pathology, National Institute of Infectious Diseases, Japan Institute for Health Security, Tokyo, Japan; 3 Department of Infection Metagenomics, Research Institute for Microbial Diseases, The University of Osaka, Osaka, Japan; 4 Center for Advanced Modalities and DDS, The University of Osaka, Osaka, Japan; 5 Division of Microbiology, Department of Pathology and Microbiology, Nihon University School of Medicine, Tokyo, Japan; 6 Center for Infectious Disease Education and Research, The University of Osaka, Osaka, Japan; The University of Texas Medical Branch at Galveston, UNITED STATES OF AMERICA

## Abstract

Single-round infectious rotavirus, which lacks a gene essential for virion assembly, serves not only as a safe and effective rotavirus vaccine but also as an orally-administrable viral vector vaccine that induces mucosal immunity. Previously, we generated a single-round infectious rotavirus by partially deleting the viral VP6 gene, and demonstrated its potential as a promising vaccine platform. However, this system has several limitations; namely, low viral protein expression levels and safety concerns. Here, we addressed these challenges by introducing large deletions into the VP7 or VP4 genes, which are dispensable for viral protein expression but essential for virion assembly. These VP7- or VP4-defective viruses exhibited markedly higher protein expression in wild-type MA104 cells than the previously developed VP6-defective virus. In addition, the large deletions reduce the risk of viral reversion, thereby increasing both efficacy and safety. In a mouse model, these viruses induced neutralizing antibodies at levels comparable with those elicited by wild-type rotavirus, indicating their potential as rotavirus vaccines. Moreover, a VP4-defective rotavirus harboring a heterologous gene achieved high expression of heterologous proteins, warranting its application as a viral vector vaccine. To further increase safety, we established a reverse genetics system for the bovine rotavirus WC3 strain, a parental strain of the licensed live attenuated rotavirus vaccine, and successfully generated a single-round VP4-defective rotavirus based on the WC3 backbone. Taken together, these optimizations facilitate development of safe and effective single-round infectious rotavirus platforms suitable for human use.

## Author summary

Rotavirus remains a leading cause of acute gastroenteritis in infants worldwide, prompting continued development of safer and more effective vaccines. Single-round infectious rotavirus, which lacks a critical replication gene, not only serves as a potentially safe rotavirus vaccine but can also function as an orally-administrable viral vector vaccine. Previously, we developed such a system by deleting a viral capsid gene; however, the current system has two major drawbacks: low protein expression levels and safety concerns. Here, we aimed to overcome these obstacles by introducing large deletions into the viral surface protein genes. This strategy enabled generation of single-round infectious viruses with markedly higher protein expression and a minimal likelihood of viral reversion. Moreover, we applied the system to a non-pathogenic rotavirus vaccine strain backbone, which increases its safety profile even further. Taken together, these improvements represent significant advances in single-round infectious rotavirus technology, making it a safer and more efficient platform for both rotavirus vaccine development and vector-based immunization of humans.

## Introduction

Rotavirus is a major cause of severe diarrhea in infants [1]. While live attenuated rotavirus vaccines such as Rotarix and RotaTeq are available widely, and have reduced the global burden of rotavirus infections significantly [2–4], an estimated 128,500 infants still die each year from rotavirus infection [5]. Moreover, live attenuated vaccine strains can disseminate into the environment and may cause vaccine-derived infections [6,7]. These concerns highlight the need for further improvements to rotavirus vaccines.

Rotavirus, a member of the genus *Rotavirus* (family *Sedoreoviridae*) [8], possesses an 11-segmented double-stranded RNA (dsRNA) genome that encodes six structural proteins (VP1–4, VP6, and VP7) and six nonstructural proteins (NSP1–6) [9]. The infectious virions comprise three capsid protein layers [10], the inner, intermediate, and outer layers, formed by VP2, VP6, and VP7, respectively. VP4 exists as spike-like structures on the surface of the virion, which mediate viral attachment to susceptible cells [11]. Additionally, VP7 interacts with co-receptors such as integrins to facilitate viral entry [12]. VP7 and VP4 are highly diverse, and comprise multiple genotypes: VP7 defines G genotypes while VP4 defines P genotypes [9]. Because VP7 and VP4 are exposed on the virion surface, they serve as major targets for neutralizing antibodies [13]. Once rotavirus enters host cells, the outer layer proteins are removed, and viral mRNAs are transcribed from dual-layered particles, leading to viral protein translation. The structural proteins VP1, VP2, VP3, and VP6 contribute to RNA transcription and replication [14–17]. The nonstructural proteins NSP2 and NSP5 are essential for forming inclusion bodies (termed viroplasms), within which new viral particles assemble and dsRNA synthesis occurs [18].

A single-round infectious rotavirus that lacks a gene essential for virion assembly is a promising vaccine platform. Previously, we developed a single-round infectious rotavirus by partially mutating the VP6 gene [19]. This system offers multiple benefits: (1) no dissemination of infectious virus into the environment, (2) it is amenable to oral administration, and (3) it can be used as a viral vector vaccine that stimulates mucosal immunity. Despite these advantages, several limitations remain. First, because VP6 plays a role in viral RNA transcription and replication, mutating part of this gene reduces viral protein expression (possibly through impaired viral RNA transcription), which could attenuate immunogenicity. Second, we mutated only six amino acid residues within VP6, meaning that viral reversion remains possible. Finally, the highly replicative simian rotavirus SA11 strain was used as the backbone virus, raising concerns about pathogenicity in humans. A non-pathogenic vaccine strain would be preferable for clinical application in humans. Thus, additional improvements to this single-round infectious rotavirus system are needed.

Here, we aimed to optimize the single-round infectious rotavirus system to address these challenges. By introducing large deletions into the viral surface protein genes, we generated single-round infectious viruses that maintained high levels of viral protein expression while minimizing the risk of viral reversion. To increase safety further, we established a reverse genetics system for a rotavirus vaccine strain, and successfully generated a VP4-defective single-round infectious rotavirus vaccine strain. These advancements support development of safe and effective single-round infectious rotavirus platforms suitable for use in humans.

## Results

### Generation of VP7-defective single-round infectious rotavirus

Previously, we developed a single-round infectious rotavirus by partially mutating the VP6 gene segment [19]; however, because VP6 contributes to transcription and replication of viral RNA, these mutations reduced expression of viral proteins. To maintain robust protein production while at the same time preventing virion assembly, we chose to delete viral proteins that are presumably not required for RNA replication. In this study, we focused on the major surface proteins, VP7 (outer capsid) and VP4 (spike) [11,12].

**Fig 1:**
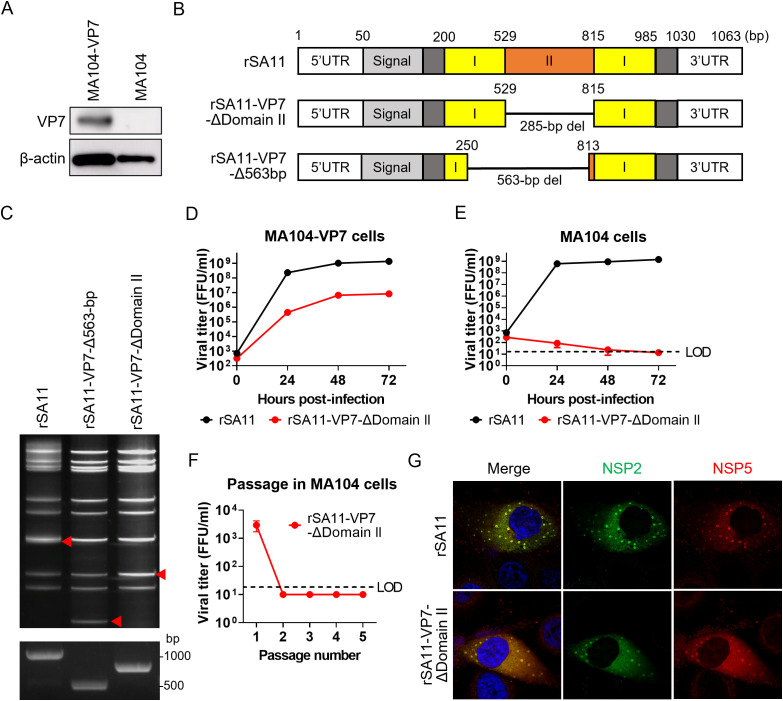
Generation of VP7-defective single-round infectious rotavirus. (A) Western blot analysis of VP7 expression by MA104-VP7 cells. (B) Schematic representation of the VP7 domain, with the nucleotide positions indicated above. Domain I and II are shown in yellow and orange, respectively. The “Signal” indicates signal sequence of VP7, shown in light gray. Disordered regions were shown in dark gray. (C) Verification of VP7 gene deletions. The top panel shows dsRNA electrophoresis of recombinant viruses; the bottom panel shows PCR amplification of the VP7 segment. The red triangles indicate the position of the VP7 segments. (D, E) Replication kinetics of rSA11 and rSA11-VP7-ΔDomain II in MA104-VP7 cells (D) and standard MA104 cells (E). Cells were infected with viruses at an MOI of 0.01, and harvested at the indicated times. The limit of detection (LOD) was 20 FFU/mL. Titers below this threshold were plotted as half the LOD (10 FFU/mL). (F) Serial passage of rSA11-VP7-ΔDomain II in MA104 cells. MA104 cells were infected with rSA11-VP7-ΔDomain II at an MOI of 1.0 and incubated for 7 days. Following freeze-thaw lysis, 10% of the lysate was transferred to fresh MA104 cells. This process was repeated five times, and viral titers were measured at each passage. (G) Viroplasm formation. MA104 cells were infected with rSA11 or rSA11-VP7-ΔDomain II at an MOI of 1.0, fixed at 8 h post-infection, and stained to detect NSP2 and NSP5.

First, we aimed to generate a VP7-defective single-round infectious rotavirus using the reverse genetics system of the simian rotavirus SA11 strain, a highly replicative laboratory strain [20]. The recombinant SA11 strain, designated rSA11, served as a wild-type virus. To support propagation of VP7-defective viruses, we used a lentivirus vector system to generate MA104 cells stably expressing SA11 VP7 (MA104-VP7; Fig 1A) essentially as described previously [19]. The VP7 protein is divided structurally into several regions: a signal sequence, the Rossmann-fold domain (domain I), the β-barrel domain (domain II), and disordered regions (Fig 1B) [21]. Both domain I and domain II are essential for VP7 trimer formation in the presence of Ca² ⁺ ions, a process critical for virion assembly. Using MA104-VP7 and the reverse genetics system for the SA11 strain, we rescued two VP7-defective rotaviruses: rSA11-VP7-ΔDomain II, which lacks domain II, and rSA11-VP7-Δ563-bp, carrying a 563-bp deletion in the VP7 segment, which leaves only 250-bp at both the 5’ and 3’ ends (Fig 1B). The gene deletions were confirmed by dsRNA electrophoresis and PCR amplification of the entire VP7 segment (Fig 1C). Because the rSA11-VP7-ΔDomain II yielded higher viral titers than rSA11-VP7-Δ563-bp (S1 Fig), we focused on the former. The rSA11-VP7-ΔDomain II was able to replicate efficiently in MA104-VP7 cells, but not in standard MA104 cells (Fig 1D and 1E). Repeated passage in wild-type MA104 cells did not yield any infectious virions (Fig 1F), confirming its single-round infectivity. Notably, the rSA11-VP7-ΔDomain II induced formation of viroplasm in infected cells (Fig 1G), indicating robust expression of viral proteins despite deletion of VP7.

### Generation of VP4-defective single-round infectious rotavirus

**Fig 2:**
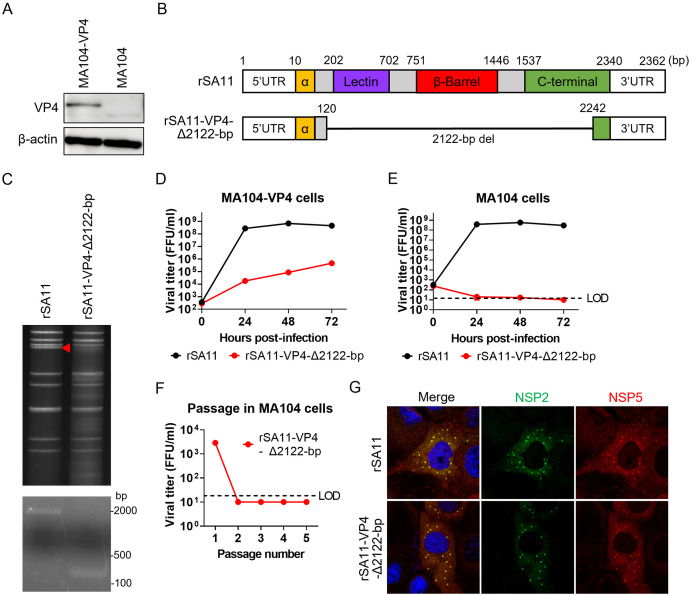
Generation of VP4-defective single-round infectious rotavirus. (A) Western blot analysis of VP4 expression by MA104-VP4 cells. (B) Schematic representation of the VP4 domain, with nucleotide positions indicated above. The α domain, lectin domain, β-barrel domain, and C-terminal domain are shown in orange, purple, red, and green, respectively. Disordered regions are shown in gray. (C) Verification of VP4 gene deletion. The top panel shows dsRNA electrophoresis of recombinant viruses; the bottom panel shows PCR amplification of the VP4 segment. The red triangle indicates the position of the VP4 segment. (D, E) Replication kinetics of rSA11 and rSA11-VP4-Δ2122-bp in MA104-VP4 cells (D) and standard MA104 cells (E). Cells were infected with viruses at an MOI of 0.01 and harvested at the indicated times. The limit of detection (LOD) was 20 FFU/mL. Titers below this threshold were plotted as half the LOD (10 FFU/mL). (F) Serial passage of rSA11-VP4-Δ2122-bp in MA104 cells. MA104 cells were infected with rSA11-VP4-Δ2122-bp at an MOI of 1.0 and incubated for 7 days. Following freeze-thaw lysis, 10% of the lysate was transferred to fresh MA104 cells. This process was repeated five times, and viral titers were measured at each passage. (G) Viroplasm formation. MA104 cells were infected with rSA11 or rSA11-VP4-Δ2122-bp at an MOI of 1.0, fixed at 8 h post-infection, and stained to detect NSP2 and NSP5.

In addition, we attempted to generate VP4-defective single-round infectious rotavirus. To support propagation of VP4-defective viruses, we again used a lentivirus vector system to generate MA104 cells stably expressing SA11 VP4 (MA104-VP4; Fig 2A). VP4 is divided structurally into the α domain, a lectin domain, a β‐barrel domain, a C‐terminal domain, and disordered regions, all of which are presumably important for viral attachment and entry (Fig 2B) [10]. We rescued a virus carrying a 2122-bp deletion in the VP4 segment, retaining only 120-bp at both the 5′ and 3′ ends (rSA11-VP4-Δ2122-bp; Fig 2B). The gene deletion was confirmed by dsRNA electrophoresis and PCR amplification of the entire VP4 segment (Fig 2C). The rSA11-VP4-defective virus was able to replicate efficiently in MA104-VP4 cells, but not in standard MA104 cells (Fig 2D and 2E). Repeated passage in wild-type MA104 cells did not yield any infectious virions (Fig 2F). Nevertheless, rSA11-VP4-Δ2122-bp induced formation of viroplasm in infected cells (Fig 2G). Taken together, these findings indicate that rSA11-VP4-Δ2122-bp displays a single-round replication phenotype similar to that of rSA11-VP7-ΔDomain II.

### Comparison of protein expression by single-round infectious rotaviruses

Previously, we generated a VP6-defective single-round infectious rotavirus (rSA11-VP6-169-174_Linker_, referred to hereafter as rSA11-VP6-defective), which exhibits lower expression of viral proteins in MA104 cells than the wild-type rSA11 strain [19]. To determine whether our newly developed VP7- and VP4-defective single-round infectious viruses express proteins at higher levels, we compared them with rSA11-VP6-defective. For clarity, rSA11-VP7-ΔDomain II is designated rSA11-VP7-defective, and rSA11-VP4-Δ2122-bp is referred to as rSA11-VP4-defective.

**Fig 3:**
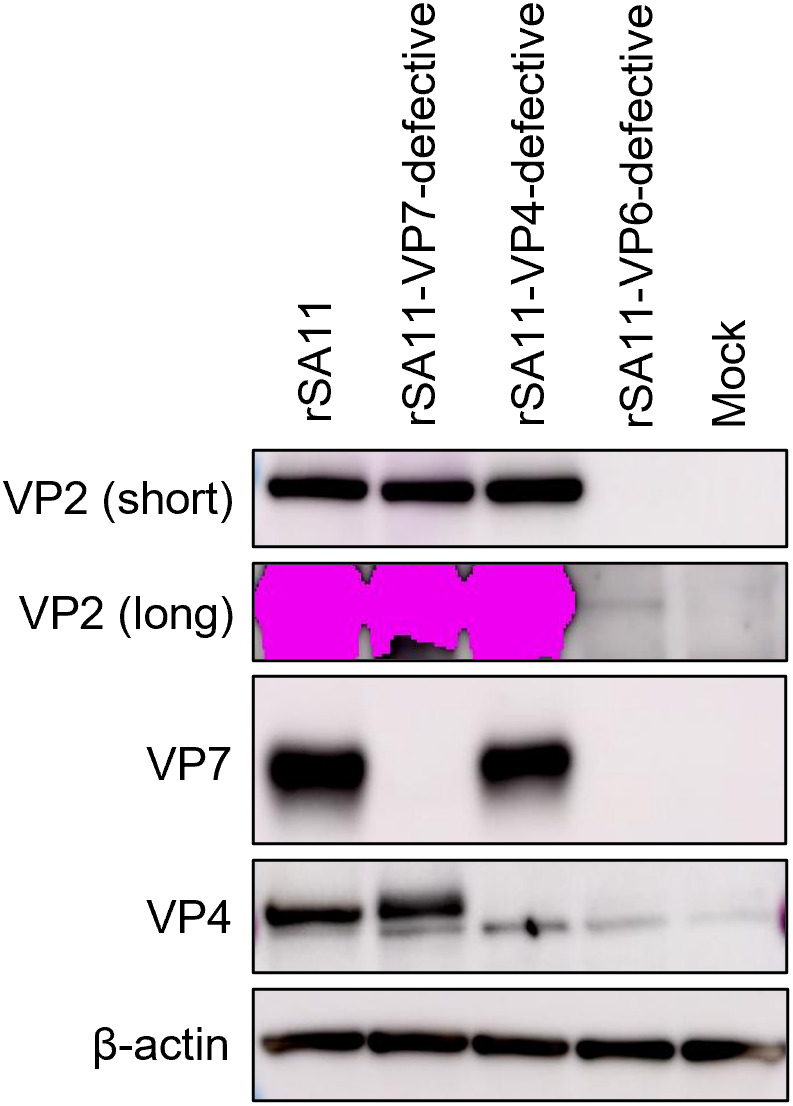
Comparison of protein expression by single-round infectious rotaviruses. Expression of viral proteins in infected cells. MA104 cells were infected with viruses at an MOI of 1.0 and cultured for 16 h in DMEM supplemented with 5% FBS.

The rSA11-VP6-defective virus exhibited markedly lower protein expression in MA104 cells, a finding consistent with previous observations (Fig 3) [19]. Expression of VP2 was barely detectable even after a long exposure time. By contrast, the rSA11-VP7-defective and rSA11-VP4-defective variants showed high expression of VP2; indeed, levels were similar to those observed for wild-type rSA11. In addition, rSA11-VP7-defective and rSA11-VP4-defective lacked expression of VP7 and VP4, respectively, confirming deletion at the protein level. Collectively, these findings suggest that viruses lacking VP7 or VP4 maintain robust protein expression, which may support potent immune activation.

### Generation of VP4-defective single-round infectious rotavirus expressing a heterologous gene

**Fig 4:**
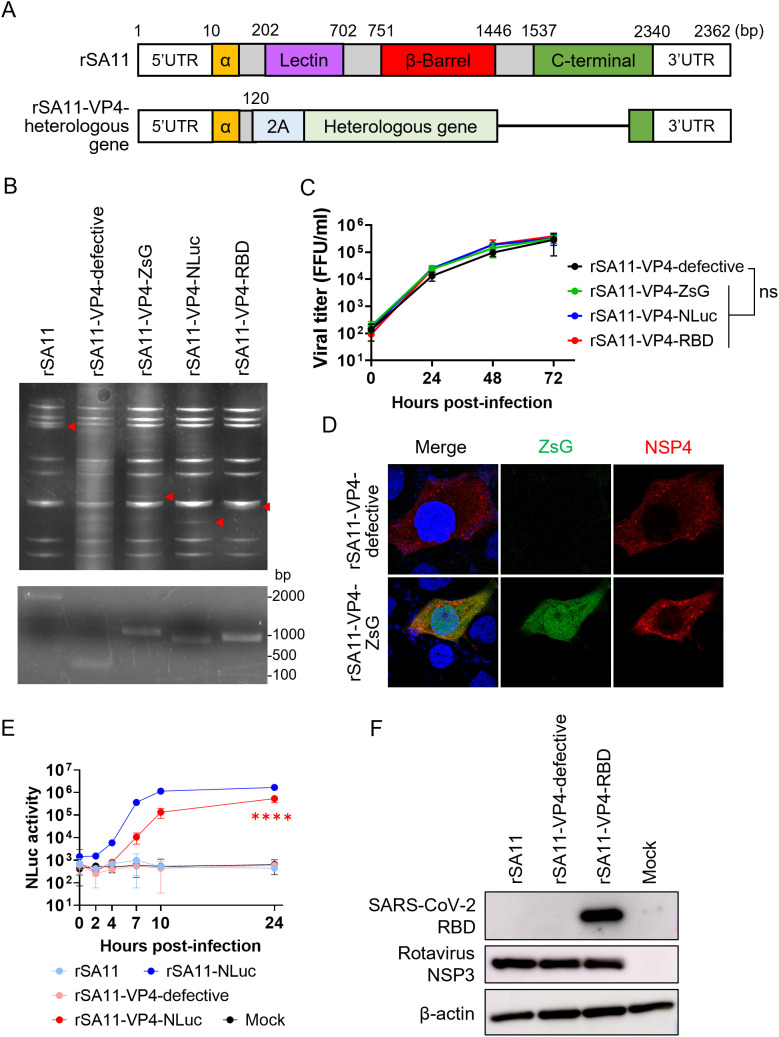
Generation of VP4-defective single-round infectious rotaviruses expressing a heterologous gene. (A) Schematic of the VP4 domain structure. “2A” indicates the porcine teschovirus 2A peptide gene. Nucleotide positions are indicated above the bar representation. (B) Confirmation of heterologous gene insertion. The top panel shows dsRNA electrophoresis from recombinant viruses; the bottom panel shows PCR amplification of the VP4 segment. The red triangles indicate the position of the VP4 segment from wild-type rSA11 or mutant rSA11 carrying the heterologous gene (ZsG, NLuc, or SARS-CoV-2 RBD). (C) Growth kinetics of recombinant rotaviruses expressing a heterologous gene. MA104-VP4 cells were infected with viruses at an MOI of 0.01 and harvested at designated times. Growth of viruses expressing heterologous genes was compared with that of rSA11-VP4-defective at 72 h post-infection. Statistical significance was analyzed by one-way ANOVA with Dunnett’s post hoc test (comparing rSA11-VP4-defective with the other viruses). P < 0.05 was regarded as significant. ns = not significant. (D–F) Verification of heterologous gene expression. (D) Expression of ZsG, as detected in an immunofluorescence assay. MA104 cells were infected with rSA11-VP4-defective or rSA11-VP4-ZsG at an MOI of 1.0 and fixed at 8 h post-infection. (E) NLuc kinetics. MA104 cells were infected with viruses at an MOI of 1.0 and cultured in DMEM supplemented with 5% FBS for the designated times. The statistical significance of rSA11-NLuc and rSA11-VP4-NLuc expression at 24 h post-infection was evaluated using one-way ANOVA with Dunnett’s post hoc test (comparing rSA11-NLuc with the other viruses); P < 0.05 was considered significant. ****P < 0.05. (F) Expression of SARS-CoV-2 RBD confirmed by Western blot analysis. MA104 cells were infected with viruses at an MOI of 1.0 and harvested at 16 h post-infection.

The single-round infectious rotavirus system is useful not only as a rotavirus vaccine but also as a viral vector vaccine. Improved protein expression levels indicate its suitability as a viral vector vaccine; therefore, we inserted heterologous genes into the viral genome. The NSP1 and NSP3 segments are often used for heterologous gene insertion [22,23]. Here, we chose the VP4 segment for heterologous gene insertion because the large deletion in the VP4-defective single-round infectious virus creates space that may accommodate a heterologous gene, as demonstrated for other viruses (Fig 4A) [24,25]. Moreover, successful insertion into the VP4 segment could expand the potential of the rotavirus vector system by enabling expression of multiple heterologous genes in combination with the NSP1 and/or NSP3 segments.

We successfully generated three VP4-defective variants: rSA11-VP4-ZsG (carrying ZsGreen), rSA11-VP4-NLuc (carrying NanoLuc luciferase), and rSA11-VP4-RBD (carrying the SARS-CoV-2 receptor-binding domain). Successful insertion of the heterologous gene was confirmed by dsRNA electrophoresis and PCR (Fig 4B). The recombinant viruses exhibited robust viral replication in MA104-VP4 cells, which was comparable with that of rSA11-VP4-defective (Fig 4C). Expression of the heterologous gene was confirmed in MA104 cells (Fig 4D–4F). While the luciferase activity of rSA11-VP4-NLuc was lower than that of replication-competent rSA11 expressing NLuc (rSA11-NLuc), the difference at 24 h post-infection was only about 3-fold (Fig 4E). By contrast, the difference between rSA11-NLuc and the VP6-defective virus expressing NLuc was about 50-fold [19]. Thus, the VP4-defective single-round infectious virus system could serve as a promising viral vector vaccine platform.

### Pathogenicity of rSA11-VP7-defective and rSA11-VP4-defective in mice

**Fig 5:**
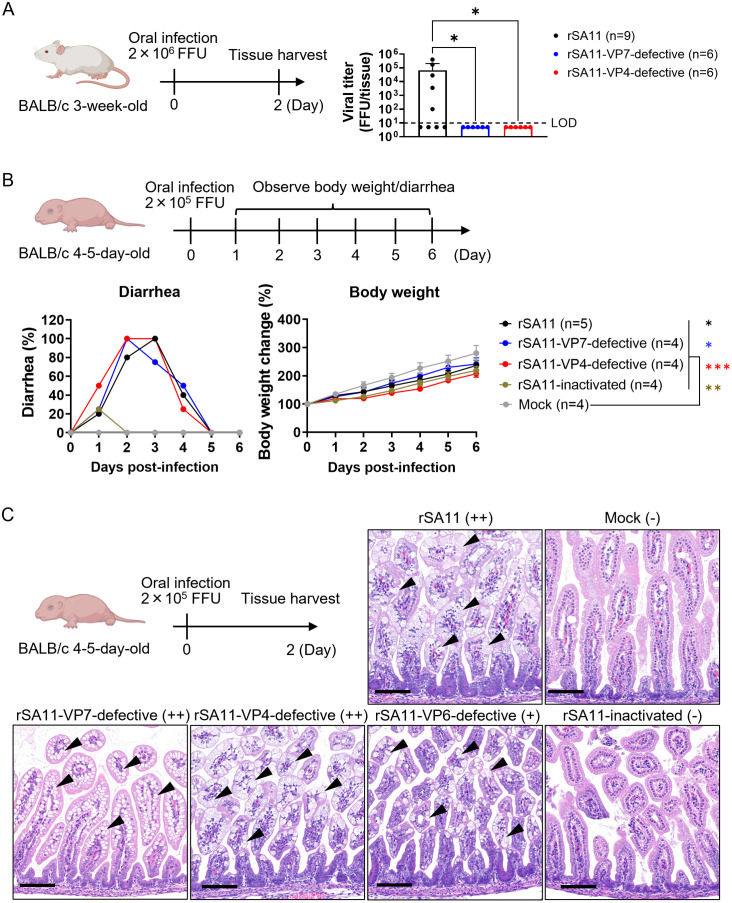
Pathogenicity of single-round infectious rotaviruses. (A) Viral replication in intestines. Three-week-old BALB/c mice (n = 6–9 per group) were orally infected with 2 × 10⁶ FFU of virus. Cecum tissue samples were collected at 2 days post-infection, homogenized, and subjected to viral titration assays. The limit of detection (LOD) was 10 FFU/tissue; values below this were plotted at 5 FFU/tissue. Statistical significance was evaluated using the Kruskal-Wallis test. ns = not significant; *P < 0.05. (B) Pathogenicity analysis. Four- or five-day-old BALB/c mice (n = 4–5 per group) were orally infected with 2 × 10⁵ FFU of virus. The left graph shows the percentage of mice with diarrhea in each group. The right graph shows body weight changes following infection. The body weight of virus-infected mice was compared with that of mock-infected mice at 6 days post-infection. Statistical significance was evaluated using one-way ANOVA with Dunnett’s post hoc test (comparing Mock with the other groups). ns = not significant; *P < 0.05; **P < 0.01; ***P < 0.001. (C) Histopathological analysis of the small intestine. Four- or five-day-old BALB/c mice were infected with 2 × 10⁵ FFU of the virus. At 2 days post-infection, jejunum tissues were collected and stained with hematoxylin and eosin (HE) staining. Black arrowheads indicate representative points of vacuolar degeneration. Histopathological changes were graded as follows: ++ = vacuolization in >50% of enterocytes, + = vacuolization in 1–50% of enterocytes, – = no vacuolization. The grading is indicated in parentheses next to each sample name. Scale bars indicate 100 µm. Images of mice were created with BioRender.com.

We would not expect a single-round infectious rotavirus to produce progeny virions in the mouse intestine. To verify this, we infected 3-week-old BALB/c mice with either rSA11-VP7-defective or rSA11-VP4-defective virus, with the wild-type rSA11 strain serving as a control. Infectious rSA11 was detected in cecum samples harvested at 2 days post-infection (Fig 5A). By contrast, no infectious rSA11-VP7-defective or rSA11-VP4-defective virions were detected, confirming that these single-round infectious viruses are unable to produce progeny virions *in vivo*.

To verify whether reduced viral replication translates into lower pathogenicity, we infected neonatal BALB/c mice with either rSA11-VP7-defective or rSA11-VP4-defective viruses, with rSA11 and UV-inactivated rSA11 (referred to hereafter as rSA11-inactivated) serving as controls. Diarrhea and body weight were monitored for 6 days post-infection. Consistent with observations for the rSA11-VP6-defective virus [19], both rSA11-VP7-defective and rSA11-VP4-defective caused diarrhea and growth retardation (Fig 5B). Intriguingly, rSA11-inactivated also caused weight loss and mild diarrhea (Fig 5B). When comparing rSA11-inactivated with the single-round infectious viruses, we noted a clear difference with respect to diarrhea, but not with respect to body weight loss. This finding suggests that even a single round of infection is sufficient to induce diarrhea, whereas body weight loss may be caused by the presence of viral particles.

To assess pathogenicity further, we examined the jejunum tissues of infected mice by hematoxylin and eosin (HE) staining (Fig 5C). Tissues were collected at 2 days post-infection; therefore, vacuolar degeneration was the only apparent change (no infiltration of the lamina propria or crypt hyperplasia were observed). Therefore, pathological changes were graded as follows: ++ = vacuolization in >50% of enterocytes, + = vacuolization in 1–50% of enterocytes, – = no vacuolization. rSA11 induced clear vacuolization, whereas rSA11-inactivated and mock control did not (Fig 5C). All of the single-round infectious rotaviruses, including rSA11-VP6-defective, induced vacuolar changes. rSA11-VP6-defective caused slightly milder vacuolization than the others, consistent with its attenuated expression of viral proteins (Fig 3). There were no apparent differences between the wild-type rSA11 and the other single-round infectious rotaviruses (rSA11-VP7-defective and rSA11-VP4-defective): in both cases more than 50% enterocytes showed vacuolar degeneration. Taken together, the data suggest that rSA11-VP7-defective and rSA11-VP4-defective exhibit a pathogenicity similar to that of rSA11 in neonatal mice, which is consistent with a prior study of rSA11-VP6-defective [19].

### Immunogenicity of rSA11-VP7-defective and rSA11-VP4-defective in mice

**Fig 6:**
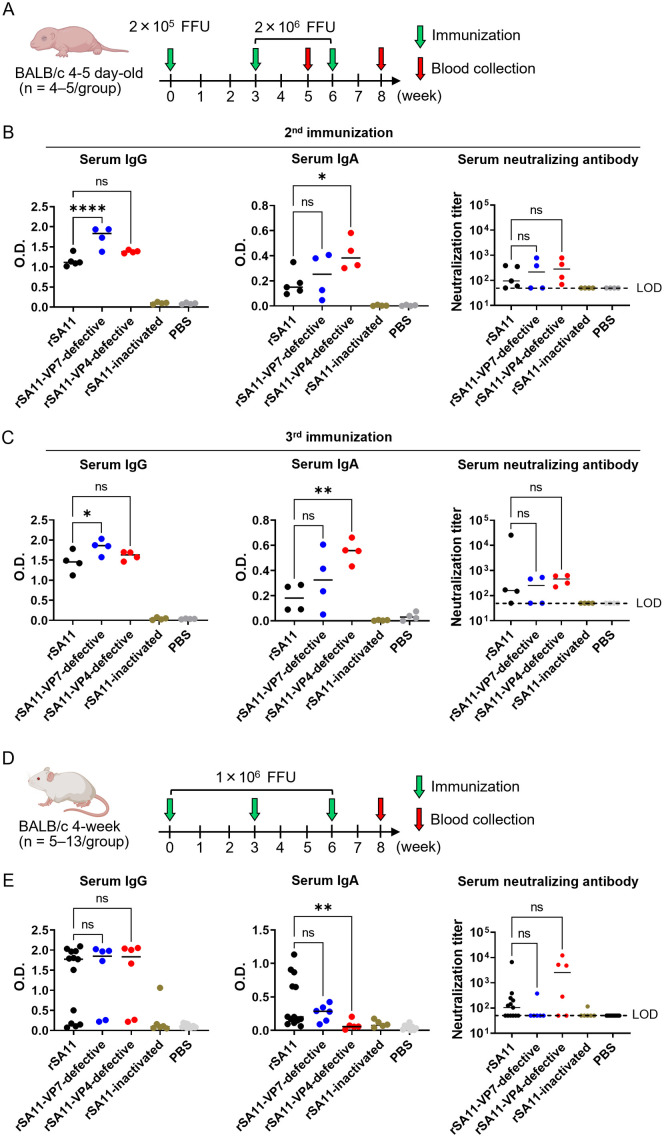
Immunogenicity of single-round infectious rotaviruses. (A) Protocol used to immunize neonatal mice. Four- or five-day-old BALB/c pups (n = 4–5/group) were orally immunized (three times at 3-week intervals). Blood was obtained 2 weeks after the second and third immunizations. (B, C) Rotavirus-specific antibody responses following the (B) second and (C) third immunizations. Serum IgG and IgA were measured by ELISA. Antibody levels in mice immunized with single-round infectious rotavirus were compared with those in mice immunized with wild-type virus. One-way ANOVA with Dunnett’s post hoc test (comparing rSA11 with the other groups) was used for statistical analysis. Neutralization titers were measured using HiBiT-expressing rSA11; the limit of detection (LOD) was 1:50. Samples below 1:50 were plotted at 1:50. The Kruskal-Wallis test was used for statistical analysis. P < 0.05 was regarded as significant. ns = not significant; *P < 0.05; **P < 0.01; ****P < 0.0001. (D) Immunization of 3-week-old BALB/c mice (n = 5–13/group). Oral immunization was repeated three times at 3-week intervals. (E) Levels of rotavirus-specific antibodies after the third immunization. Images of mice were created with BioRender.com.

To evaluate immunogenicity, we orally inoculated neonatal mice with rSA11-VP7-defective or rSA11-VP4-defective (Fig 6A), with rSA11 and SA11-inactivated serving as controls. The single-round infectious viruses elicited detectable levels of serum IgG, IgA, and neutralizing antibodies against rotavirus (Fig 6B and 6C). By contrast, none of these antibodies were observed in mice immunized with rSA11-inactivated, indicating that single-round infection is crucial for inducing an immune response. Although IgG and IgA levels induced by the wild-type virus and the single-round variants varied, there was no statistically significant difference in the level of neutralizing antibody induction. A similar experiment was conducted using 4-week-old BALB/c mice (Fig 6D). Again, although IgG and IgA levels varied somewhat, there was no statistically significant difference in induction of neutralizing antibodies (Fig 6E). Overall, these findings demonstrate that single-round infectious viruses are as immunogenic as wild-type rSA11 in mice.

### Improvement of single-round infectious rotavirus productivity

**Fig 7:**
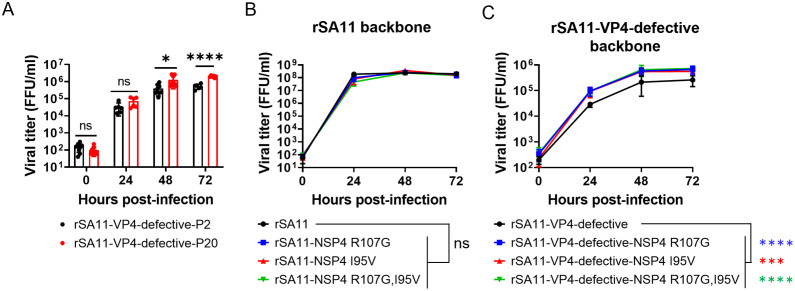
Improving the productivity of VP4-defective single-round infectious rotavirus. (A) Growth of rSA11-VP4-defective-P2 and rSA11-VP4-defective-P20. MA104-VP4 cells were infected with rSA11-VP4 defective-P2 or rSA11-VP4 defective-P20 at an MOI of 0.01 and harvested at the designated times. The results of three independent experiments are shown. A t-test was used to evaluate significance, with P < 0.05 considered significant. ns = not significant; *P < 0.05; ****P < 0.0001. (B, C) Replication of NSP4-mutant rotaviruses with (B) the rSA11 backbone or (C) the rSA11-VP4-defective backbone. MA104-VP4 cells were infected with viruses at an MOI of 0.01 and collected at the designated times. Growth of the NSP4 mutant viruses was compared with that of their parental virus at 72 h post-infection. Statistical analysis was performed with one-way ANOVA with Dunnett’s post hoc test. P < 0.05 was considered significant. ns = not significant; ***P < 0.001; ****P < 0.0001.

For clinical application, it is essential to achieve a sufficient viral yield; however, the single-round infectious rotaviruses in our system reached a peak titer of approximately 10⁶ FFU/mL, which is 100-fold lower than that of the wild-type virus. To address this limitation, we subjected rSA11-VP7-defective and rSA11-VP4-defective to 20 rounds of serial passage in MA104 cells expressing VP7 or VP4, respectively. At passage 20, the titer of rSA11-VP4-defective (designated rSA11-VP4-defective-P20) was approximately 5-fold higher than that of rSA11-VP4-defective at passage 2 (rSA11-VP4-defective-P2) (Fig 7A). By contrast, rSA11-VP7-defective did not show any marked increase in viral growth, even after 20 passages (S2 Fig). Next-generation sequencing revealed that rSA11-VP4-defective-P20 acquired R107G and/or I95V substitutions in NSP4. Introducing these mutations into rSA11 did not affect viral growth (Fig 7B); however, these mutations increased replication of rSA11-VP4-defective (Fig 7C), indicating that these amino acid substitutions contribute to improved productivity in the VP4-defective system.

### Generation of a reverse genetics system for bovine rotavirus WC3 strain and its application to single-round infectious rotavirus

Our existing single-round infectious rotavirus system was based on the simian rotavirus SA11 strain, which is a highly productive prototype strain; however, the pathogenicity of this strain in humans remains unclear. Although single-round infectious viruses are theoretically safe for clinical use, using an established rotavirus vaccine strain that is confirmed to be safe in humans would further strengthen its safety profile. Thus, we aimed to develop a single-round infectious rotavirus based on a vaccine strain.

The licensed RotaTeq vaccine comprises five human–bovine reassortant rotaviruses (WI79–9, SC2–9, WI78–8, BrB-9, and WI79–4), each containing a human rotavirus VP7 or VP4 segment within a bovine rotavirus WC3 strain backbone (G6P[5] genotype) [2,26,27]. Specifically, the WI79–9, SC2–9, WI78–8, BrB-9, and WI79–4 strains possess the G1, G2, G3, G4, and P[8] genotypes, respectively, from human rotavirus in a WC3 backbone [26]. In addition, the WI79–9 and SC2–9 strains carry the VP3 segment from human rotavirus [26].

**Fig 8:**
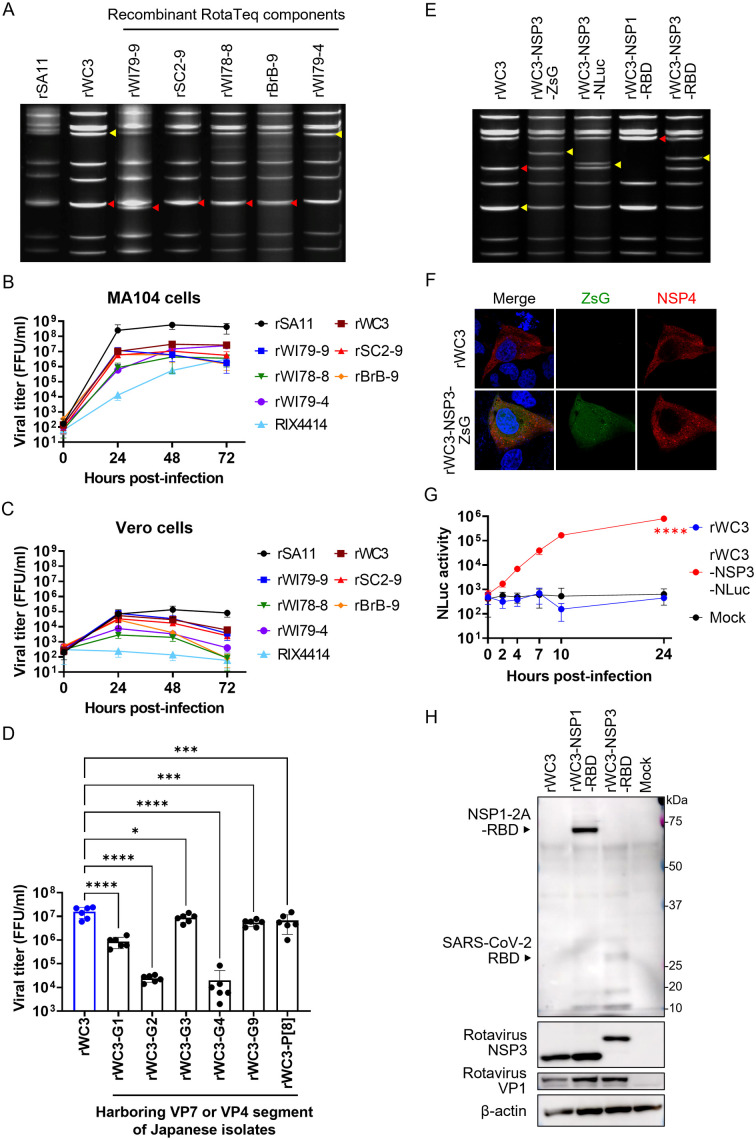
Establishment of reverse genetics system for a rotavirus vaccine strain. (A) dsRNA electrophoresis of the recombinant RotaTeq component strains. The strains rWI79-9, rSC2-9, rWI78-8, rBrB-9, and rWI79-4 harbor human rotavirus VP7 or VP4 segments corresponding to G1, G2, G3, G4, and P[8], respectively, within the WC3 strain backbone. The red and yellow triangles indicate the positions of the VP7 and VP4 segments, respectively. (B, C) Growth curves of the recombinant RotaTeq component strains. (B) MA104 or (C) Vero cells were infected with viruses at an MOI of 0.01, and the cells were harvested at the designated time points. (D) Growth of the rWC3 strain harboring human rotavirus VP7 or VP4 segment from Japanese isolates. MA104 cells were infected with viruses at an MOI of 0.001 and then harvested at 72 h post-infection. The G1, G2, G3, and G9 genotypes were derived from human rotavirus Japanese clinical isolate strains U14, U6, U4, and U8, respectively. The G4 and P[8] genotypes were derived from the human rotavirus Odelia strain. Statistical significance was evaluated using one-way ANOVA with Dunnett’s post hoc test (comparing rWC3 with the other viruses). *P < 0.05; ***P < 0.001; ****P < 0.0001. (E) dsRNA electrophoresis of recombinant rotaviruses harboring heterologous genes. The red and yellow triangles indicate the position of the NSP1 and NSP3 segments, respectively. (F–H) Validation of heterologous gene expression. (F) Detection of ZsG by IFA. MA104 cells were infected with rWC3 or rWC3-NSP3-ZsG at an MOI of 1.0 and fixed at 8 h post-infection. (G) NLuc kinetics. MA104 cells were infected with rWC3 or rWC3-NSP3-NLuc at an MOI of 1.0 and cultured in DMEM supplemented with 5% FBS for the designated times. Statistical significance between rWC3 and rWC3-NLuc at 24 h post-infection was evaluated using one-way ANOVA with Dunnett’s post hoc test (comparing rWC3 with the other groups). P < 0.05 was considered significant. ****P < 0.05. (H) Expression of SARS-CoV-2 RBD confirmed by Western blotting. MA104 cells were infected with viruses at an MOI of 1.0 and harvested at 16 h post-infection.

The parental WC3 strain is not pathogenic to humans [27]. Thus, we synthesized the genomic cDNA of the WC3 strain using the consensus sequences of the five RotaTeq component strains, and successfully rescued a recombinant WC3 strain (rWC3) (Fig 8A). We also generated recombinant rotaviruses harboring VP7, VP4, and/or VP3 segments from RotaTeq component strains in the WC3 backbone, thereby reproducing the RotaTeq component strains (designated rWI79–9, rSC2–9, rWI78–8, rBrB-9, and rWI79–4; Fig 8A). These recombinant strains replicated efficiently in both MA104 and Vero cells, although their titers remained lower than those of rSA11 (Fig 8B and 8C). Notably, growth of rWC3 was superior to that of a RIX4414 strain (a Rotarix vaccine strain; G1P[8] human rotavirus) in both cell lines (Fig 8B and 8C). Additionally, rWC3 monoreassortant viruses harboring the VP7 or VP4 segment from human rotavirus Japanese clinical isolates or a laboratory strain were rescued successfully (Fig 8D).

Insertion of heterologous genes (ZsG, NLuc, or SARS-CoV-2 RBD) at the end of NSP1 or NSP3 genes, linked via the porcine teschovirus 2A peptide sequence (2A), was achieved successfully on the WC3 backbone (Fig 8E) [22,23]. Expression of the heterologous gene by these recombinant strains was confirmed (Fig 8F–8H), although cleavage of NSP1-2A-RBD at the 2A peptide site appeared incomplete (Fig 8H). These findings demonstrate the utility and robustness of our WC3-based reverse genetics system.

**Fig 9:**
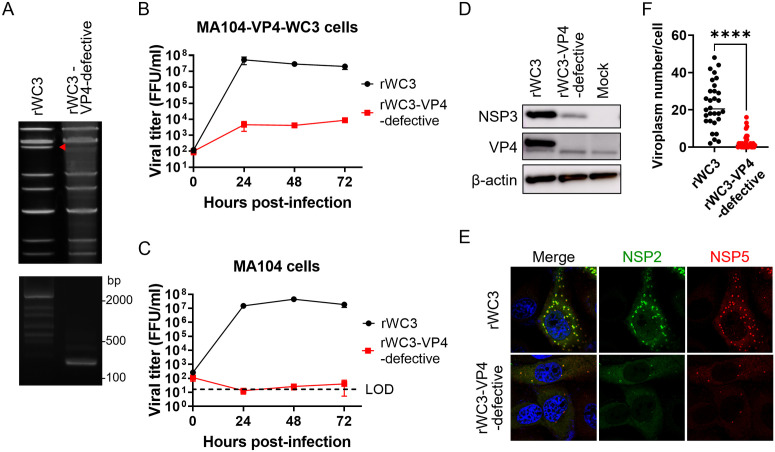
Generation of VP4-defective single-round infectious virus using the WC3 strain backbone. (A) dsRNA pattern of rWC3 and rWC3-VP4-defective. The red triangle indicates the position of the VP4 segment. (B, C) Growth of rWC3 and rWC3-VP4-defective in (B) MA104-VP4-WC3 and (C) MA104 cells. Cells were infected with rWC3 or rWC3-VP4-defective at an MOI of 0.01, and samples were harvested at the designated times. The limit of detection (LOD) was 20 FFU/mL. Titers below this threshold were plotted as half the LOD (10 FFU/mL). (D) Expression of viral proteins in infected cells. MA104 cells were infected with rWC3 or rWC3-VP4-defective at an MOI of 1.0 and cultured for 16 h in DMEM supplemented with 5% FBS. (E) Viroplasm formation. MA104 cells were infected with rWC3 or rWC3-VP4-defective at an MOI of 0.1, fixed at 8 h post-infection, and stained to detect NSP2 and NSP5. (F) Viroplasm number. The number of viroplasms (punctate structures stained with NSP2 and NSP5) in each infected cell was counted manually. Statistical significance was evaluated using Mann-Whitney test. ****P < 0.0001.

Finally, we aimed to establish a single-round infectious WC3 strain by deleting the VP4 gene. MA104 cell stably expressing VP4 of the WC3 strain (MA104-VP4-WC3) was generated using lentiviral vector system and used to rescue a VP4-defective WC3 strain harboring a 2122-bp deletion (with 120 bp remaining at each the 5′ and 3′ ends), designated rWC3-VP4-defective (Fig 9A). rWC3-VP4-defective was able to replicate in MA104-VP4-WC3 cells, but not in standard MA104 cells (Fig 9B and 9C), indicating that rWC3-VP4-defective is a single-round infectious virus. Unlike the SA11 backbone, rWC3-VP4-defective expressed viral proteins in MA104 cells at markedly lower levels than wild-type rWC3 (Fig 9D). Although rWC3-VP4-defective induced viroplasm formation in infected cells, the number of viroplasms was lower than that observed in rWC3-infected cells (Fig 9E and 9F). While further optimization is needed, the data demonstrate that we successfully established a single-round infectious rotavirus vaccine strain.

## Discussion

Although the single-round infectious rotavirus system is a promising vaccine platform, our previous system, based on partial mutation of VP6, had several drawbacks: (1) low protein expression levels, (2) the possibility of viral reversion, and (3) the use of a simian rotavirus backbone [19]. Here, we established an optimized single-round infectious rotavirus system by deleting the viral surface proteins VP7 or VP4. Viral protein expression by the VP7-defective or VP4-defective single-round infectious viruses was comparable with that of the wild-type virus, possibly through proper viroplasm formation and secondary transcription, at least in the SA11 backbone (Figs 1G, 2G and 3) [28]. In addition, the VP4-defective virus harbors a 2122-bp deletion in the VP4 segment (Fig 2B), which minimizes the chance of viral reversion. Furthermore, the VP4-defective system is also applicable to the WC3 strain backbone (Fig 9). These improvements provide a foundation for designing safe and effective single-round infectious rotavirus systems applicable to humans.

In addition to practical benefits, the rSA11-VP7-defective and rSA11-VP4-defective strains enable functional analysis of VP7 and VP4. Interestingly, a large portion (2122-bp) of the VP4 segment was deleted, indicating that inter-segment interactions involving the VP4 segment may depend primarily on the 120-bp sequences at the 5′ and 3′ termini. Further analyses to investigate the viral packaging signals or inter-segment interactions of the VP4 segment would be of interest.

Our findings demonstrate that deleting the VP7 or VP4 proteins does not reduce expression of viral proteins in the SA11 backbone markedly, although it does in the WC3 backbone (Figs 3 and 9). rWC3-VP4-defective exhibited lower levels of protein expression and fewer viroplasms than wild-type rWC3 (Fig 9D–9F), suggesting that the function of VP4 may differ between strains, and that WC3 might rely on VP4 for proper viroplasm formation. Further investigations and improvements are warranted if we are to develop single-round infectious WC3 constructs with higher levels of protein expression.

Although expression of viral protein by rSA11-VP4-defective was comparable with that by rSA11 (Fig 3), heterologous protein expression level of rSA11-VP4-NLuc was slightly lower than that of the replication-competent rSA11-NLuc (Fig 4E). Insertion of a heterologous sequence in VP4 would likely not prevent viral replication in the context of viral packaging [29,30]. We speculate that these differences in protein expression level may be due to the viral segments harboring the heterologous gene. The NLuc gene is inserted into the VP4 segment in rSA11-VP4-NLuc, whereas it is located in the NSP1 segment in rSA11-NLuc [22]. The abundance of each viral segment and its encoded product varies in infected cells, with the NSP1 segment being more abundant than the VP4 segment [31]. Further analyses incorporating heterologous genes not only into the VP4 segment but also into the NSP1 and NSP3 segments would be of interest to establish a viral vector system capable of expressing two or three heterologous proteins at higher levels [22,44].

A recent report indicates that inactivated rotavirus induces diarrhea by stimulating interferon lambda [32]. Based on this finding, one possible hypothesis is that single-round infectious virus has properties similar to those of inactivated virus in terms of pathogenicity and immunogenicity; however, our results clearly show that the single-round infectious virus induced more severe diarrhea and substantially higher levels of neutralizing antibodies than the inactivated virus (Figs 5B, 6B, 6C and 6E). This suggests that the virus can infect mice and express viral proteins in the intestine. In addition, intracellular expression of viral proteins is important for effective antibody induction. Furthermore, protein expression from single-round infection, especially NSP4, still contributes to diarrhea and pathogenicity in neonatal mice [33].

Initially, we expected to find that VP7- or VP4-defective rotavirus systems would not induce robust neutralizing antibodies against rotavirus because of the lack of intracellular expression of these proteins. Nevertheless, both the rSA11-VP7-defective and rSA11-VP4-defective viruses induced robust neutralizing antibodies at levels comparable with those induced by rSA11, at least in the mouse model (Fig 6). Our previous study suggests that simian rotavirus does not replicate efficiently in mice; as a result, even the wild-type virus induced only a modest immune response, leading to antibody levels similar to those induced by the single-round infectious viruses [19]. Other groups also reported that simian rotavirus replication was reduced in mice [34–40]. If a simian model that supports efficient replication of wild-type rSA11 was used, differences in antibody induction between the wild-type and VP7- or VP4-defective viruses might become more apparent, and induction of neutralizing antibodies by the defective viruses could be lower than that by the wild-type virus. If that is the case, VP7- or VP4-defective rotaviruses may be better suited for use as viral vector vaccines than as rotavirus vaccines in humans.

Another possibility is that antibodies targeting VP4 or VP7 domain II contribute little to neutralization. We reported previously that VP4 does not affect neutralization susceptibility significantly, whereas VP7 domain I is a major determinant of neutralization susceptibility [41]. Because both rSA11-VP4-defective and rSA11-VP7-defective viruses retain VP7 domain I, induction of neutralizing antibodies may not be reduced significantly (Fig 6). It is important to note that the neutralization assays in our studies were performed using MA104 cells, the standard cell line used for rotavirus research. Neutralizing antibodies specific for VP4 exhibit weaker neutralizing activity in MA104 monkey kidney epithelial cells than in HT-29 human colonic epithelial cells [42]. Therefore, it is possible that sera from rSA11-immunized mice might have higher neutralizing titers than those from rSA11-VP4-defective-immunized mice when HT-29 cells are used for the neutralization assay.

Regarding use as a viral vector vaccine in humans, pre-existing immunity is a major concern. Rotavirus vaccines are used widely across the world [2–4], and most individuals have been infected with rotavirus [1,5]. Our previous study identified VP7 genotypes that could escape human neutralizing antibodies induced by natural infection or vaccination [41]. Replacing VP7 with such genotypes in a VP4-defective single-round infectious rotavirus vector may be a promising strategy to escape pre-existing immunity. Additionally, the potentially lower induction of neutralizing antibodies by the VP7- or VP4-defective single-round infectious rotavirus vector is advantageous for viral vector applications as it may allow repeated administration.

We also developed a new reverse genetics system for the rotavirus vaccine strain WC3. Another widely used vaccine strain, RIX4414 (a component of Rotarix), already has an established reverse genetics system [43,45]. Here, we found that the rWC3 strain replicated better than the RIX4414 strain (Fig 8B and 8C), suggesting that reverse genetics for WC3 is more robust than that for RIX4414. A parallel comparison between rWC3 and recombinant RIX4414 would be of interest.

Exchange of the VP7 or VP4 segments with those from human rotavirus in the WC3 backbone was also successful (Fig 8A–8D). Previous vaccine strains (human–bovine reassortant rotaviruses; WI79–9, SC2–9, WI78–8, BrB-9, and WI79–4) were generated via plaque purification from co-infected cells with human and bovine rotaviruses, which is a time-consuming process [27]. By contrast, this reverse genetics system allows rapid production of more effective rotavirus vaccine strains containing the VP7 or VP4 segment of currently circulating human rotaviruses (Fig 8D). A potential concern is possible reassortment between the vaccine strain and circulating wild-type rotaviruses [45–47], which could lead to reversion to virulence, even in our single-round infectious virus system. Introducing mutations into the packaging signals may further increase safety by eliminating potential compatibility with human rotaviruses, thereby preventing reassortment events [48].

While single-round infectious rotavirus is a promising vaccine platform, its low production yield remains a significant limitation. Compared with rSA11, the peak titers for the rSA11-VP7-defective and rSA11-VP4-defective viruses were more than 100-fold lower (Figs 1D and 2D). To address this, we serially passaged the single-round infectious viruses (Fig 7). The rSA11-VP4-defective-P20 acquired adaptive mutations I95V and R107G in NSP4, which increased viral replication by approximately 5-fold. Initially, we expected that these mutations might be located in the VP6-binding domain of NSP4, which is involved in virion assembly [49]; however, they were actually located in the coiled-coil domain, which may affect pathogenicity [50]. Further investigation is needed to clarify the mechanism(s) by which these mutations affect replication, and whether they alter pathogenicity. Additionally, rWC3-VP4-defective exhibited markedly reduced peak viral titers, approximately 1,000-fold lower than those of rWC3 (Fig 9B and 9C), and showed attenuated expression of viral protein (Fig 9D–9F). A similar passaging strategy may improve its productivity and increase expression of viral protein.

In conclusion, we optimized single-round infectious rotavirus systems by deleting the VP7 or VP4 genes, and by employing a vaccine strain backbone. This system achieves higher protein expression, and potentially offers better safety profiles in humans than the earlier VP6-defective system. Further development and application of this system for viral vector vaccines is warranted.

## Materials and methods

### Ethics statement

All animal experiments were approved by the institutional animal ethics committee (approval number: BidouR03-10–0).

### Cells

Monkey kidney MA104 cells and human embryonic kidney HEK293T cells were cultured in Dulbecco’s modified Eagle’s medium (DMEM; Nacalai Tesque) supplemented with 5% fetal bovine serum (FBS; Gibco). MA104-VP7, MA104-VP4, and MA104-VP4-WC3 cells were cultured in DMEM supplemented with 5% FBS and 800 μg/mL G418 (Nacalai Tesque). Baby hamster kidney cells stably expressing T7 RNA polymerase (BHK-T7) were cultured in DMEM supplemented with 5% FBS and 1 μg/mL puromycin (Sigma-Aldrich). All cells were cultured at 37°C/5% CO_2_.

### Viruses

Simian rotavirus SA11 strain, bovine rotavirus WC3 strain, and human rotavirus RIX4414 strain were used in this study. Recombinant rotavirus SA11 strain was generated as described previously, and designated as rSA11 [20]. The recombinant WC3 strain was generated in the present study (see below) and designated as rWC3. The RIX4414 strain was provided by Dr. Hiroshi Ushijima, Nihon University. The recombinant VP6-defective single-round infectious rotavirus SA11 strain (rSA11-VP6-169-174_Linker_) was prepared as described [19]. The VP7-defective or VP4-defective SA11 strain and the WC3 strain were generated in the present study. Replication-competent reporter viruses expressing NLuc (rSA11-NLuc) or HiBiT-tag were prepared as described [22,51].

The viruses were propagated in MA104 cells. Viruses lacking a specific viral protein were grown in MA104 cells stably expressing the corresponding missing protein. DMEM supplemented with 0.5 μg/mL trypsin (Sigma-Aldrich) was used as the virus culture medium. Unless indicated otherwise, recombinant viruses were used at passage 2. The virus sequences were verified by Sanger DNA sequencing.

### Antibodies

Rabbit sera immunized with peptides corresponding to residues 615–633 of SA11-VP4, 158–171 of SA11-NSP4, 54–71 of SA11-NSP2, 299–312 of SA11-NSP2, 143–156 of SA11-NSP3, 10–28 of SA11-VP2, and 373–391 of SA11-VP1 were prepared (Eurofins) [52]. Additionally, guinea pig antiserum specific for amino acids 48–66 of SA11-NSP5 was used (Eurofins) [52]. A commercial rabbit anti-rotavirus VP7 antibody (MyBioSource) and a rabbit anti-SARS-CoV-2 RBD antibody (SinoBio) were also used. A mouse monoclonal β-actin antibody was purchased from Sigma-Aldrich. For the immunofluorescence assays, an NSP2 antibody targeting amino acids 299–312 was used for SA11, whereas an antibody targeting amino acids 54–71 was used for WC3.

### Plasmids

For lentiviral vector construction, the VP7 or VP4 gene of SA11 strain, or the VP4 gene of WC3 strain, were cloned into pLVSIN-CMV-Neo (Takara), yielding pLVSIN-VP7SA11, pLVSIN-VP4SA11, or pLVSIN-VP4WC3. Plasmids psPAX2 (#12260) and pCMV-VSV-G (#8454) were obtained from Addgene.

To generate recombinant SA11 rotavirus, the following viral rescue plasmids encoding each genome segment flanked by the T7 promoter and the hepatitis D virus ribozyme were generated: pT7-VP1SA11, pT7-VP2SA11, pT7-VP3SA11, pT7-VP4SA11, pT7-VP6SA11, pT7-VP7SA11, pT7-NSP1SA11, pT7-NSP2SA11, pT7-NSP3SA11, pT7-NSP4SA11, and pT7-NSP5SA11 [20]. Because no WC3 genome sequence is registered in GenBank, a consensus sequence from five RotaTeq component strains (WI79–9, SC2–9, WI78–8, BrB-9, or WI79–4 strains) was synthesized [26]; namely, pT7-VP1WC3, pT7-VP2WC3, pT7-VP3WC3, pT7-VP4WC3, pT7-VP6WC3, pT7-VP7WC3, pT7-NSP1WC3, pT7-NSP2WC3, pT7-NSP3WC3, pT7-NSP4WC3, and pT7-NSP5WC3. The consensus sequences synthesized in this study have been deposited in GenBank under accession number LC885185-LC885195. In addition, rescue plasmids containing the VP3, VP4, or VP7 segment of WI79–9, SC2–9, WI78–8, BrB-9, or WI79–4 strain were synthesized [26]. Deletions were introduced into pT7-VP7SA11, pT7-VP4SA11, or pT7-VP4WC3 by PCR-based mutagenesis. heterologous genes (ZsG, NLuc, or SARS-CoV-2 RBD) were incorporated into pT7-VP4SA11, pT7-NSP1WC3, or pT7-NSP3WC3 by in-fusion cloning. The following rescue plasmids encoding the VP7 or VP4 segment of human rotavirus Japanese clinical isolates or Odelia strain were used: pT7-VP7U14 (G1), pT7-VP7U6 (G2), pT7-VP7U4 (G3), and pT7-VP7U8 (G9), pT7-VP7Odelia (G4), and pT7-VP4Odelia (P[8]) [53,54]. Furthermore, pCAG-NSP2SA11, pCAG-NSP5SA11, pCAG-D1R, and pCAG-D12L, which encode rotavirus SA11 NSP2 or NSP5 or vaccinia virus D1R or D12L, respectively, were generated [22,55]. The VP7 or VP4 gene of the SA11 strain, or the VP4 gene of the WC3 strain, was inserted into pcDNA3.1 (pcDNA-VP7SA11, pcDNA-VP4SA11, pcDNA-VP4WC3). All plasmid constructs were verified by Sanger sequencing.

### Generation of MA104 cells stably expressing viral proteins

The VP7-SA11, VP4-SA11, or VP4-WC3 gene was transduced into MA104 cells using a lentiviral vector system. Briefly, HEK293T cells were transfected with the appropriate pLVSIN plasmid (pLVSIN-VP7SA11, pLVSIN-VP4SA11, or pLVSIN-VP4WC3), psPAX2, and pCMV-VSV-G at a ratio of 3:4:1. At 48 h post-transfection, culture supernatant containing lentiviral particles was harvested and used to infect MA104 cells. DMEM containing 5% FBS and 800 μg/mL G418 was added 48 h later. Surviving cells were subjected to clonal selection by limiting dilution. Expression of the target viral protein in each clone was confirmed by Western blotting (see below).

### Western blotting

Cells were washed three times in PBS and lysed in RIPA buffer (25 mM Tris-HCl [pH 7.4], 150 mM NaCl, 1% Nonidet P-40, 1% sodium deoxycholate, 0.1% SDS). The lysates were then combined with 2 × Laemmli sample buffer (50 mM Tris-HCl [pH 6.8], 2% SDS, 6% 2-mercaptoethanol, 10% glycerol, 0.01% bromophenol blue) and boiled for 5 min at 95°C. Proteins were resolved on a 10% precast SDS-polyacrylamide gel (ATTO) at 100V for 2 h. The proteins were transferred for 30 min onto Immobilon-P PVDF membranes (Merck) using a Trans-Blot SD Semi-Dry Transfer Cell (Bio-Rad) at 15V 400 mA. Viral proteins and β-actin were detected using appropriate primary antibodies, HRP-conjugated secondary antibodies, and the Chemi-Lumi One Ultra kit (Nacalai Tesque). Signals were visualized with an Amersham ImageQuant 800 (Cytiva).

### Generation of recombinant rotaviruses

Recombinant rSA11 was generated as described previously [20]. Additional recombinant rotaviruses were produced as described previously, with minor modifications [22,55]. Briefly, BHK-T7 cells (1 × 10⁵ cells/well) were seeded in a 12-well plate. On the next day, the cells were co-transfected with 11 rotavirus rescue plasmids (pT7-VP1SA11, pT7-VP2SA11, pT7-VP3SA11, pT7-VP4SA11, pT7-VP6SA11, pT7-VP7SA11, pT7-NSP1SA11, pT7-NSP2SA11, pT7-NSP3SA11, pT7-NSP4SA11, and pT7-NSP5SA11) plus four supporting plasmids (pCAG-NSP2SA11, pCAG-NSP5SA11, pCAG-D1R, and pCAG-D12L). For viruses harboring deletions or heterologous gene inserts, the corresponding rescue plasmids were replaced. pcDNA-VP7SA11, pcDNA-VP4SA11, or pcDNA-VP4WC3 was co-transfected when rescuing VP7- or VP4-defective viruses. In total, 15 or 16 plasmids (0.125 µg each) were transfected using TransIT-LT1 (Mirus). After 24 h, MA104 cells or MA104 cells expressing the relevant viral proteins (1.5 × 10⁵ cells/well) were added to DMEM containing 0.5 μg/mL trypsin and co-cultured for 5 days. Cells were then subjected to three freeze-thaw cycles, and 10% of the resulting lysate was transferred onto fresh MA104 monolayers (with or without the required viral protein). Following a 5-day incubation, cells were lysed again, and a portion of the lysate was used for immunofluorescence detection with an anti-NSP4 antibody to confirm successful rescue (see below).

### Virus titration

MA104 cells (1 × 10⁴ cells/well) were seeded in 96-well plates. On the following day, the cells were inoculated with serially-diluted virus samples and incubated overnight at 37°C. After fixing for 30 min with 4% formaldehyde and permeabilizing for 15 min with 0.5% Triton X-100, the cells were blocked for 1 h in PBS containing 2% FBS. They were then incubated with a primary anti-rotavirus NSP4 antibody, followed by a fluorescent-labeled secondary antibody. Each antibody was applied for 1 h at room temperature, with three PBS washes in between. Nuclei were stained using Hoechst 33342 (Thermo Fisher). Imaging was performed on an Axio Observer 7 fluorescence microscope (Zeiss), and infected foci were counted manually. Titers are expressed as focus forming units (FFU)/mL.

### Electrophoresis of dsRNA genome

Total viral RNA was extracted using Sepasol (Nacalai Tesque). The RNA was then combined with DNA loading dye (NEB) and resolved by 10% polyacrylamide gel electrophoresis (ATTO). Following electrophoresis, the gel was stained with GelRed (Biotium), and images were taken on a Printgraph Classic WSE-5400 (Atto).

### PCR amplification of rotavirus genome segments

Viral RNA was extracted using Sepasol (Nacalai Tesque) and then subjected to RT-PCR using the ReverTra Ace (Toyobo) and KOD One (Toyobo) reagents. The following primers were used to amplify the VP7 and VP4 segments: VP7 forward, 5’-ggctttaaaaagagagaatttccg-3’ (corresponding to 1^st^–24^th^ nucleotide of GenBank accession number LC178567) and VP7 reverse, 5’-ggtcacatcatacaattctaacc-3’ (1041^st^–1063^rd^ nucleotide of LC178567); and VP4 forward, 5’-ggctataaaatggcttcgctc-3’ (1^st^–21^st^ nucleotide of LC178569) and VP4 reverse, 5’-ggtcacatcctctagaaattactc-3’ (2339^th^–2362^nd^ nucleotide of LC178569). Amplicons were visualized with GelRed (Biotium) and imaged via a Printgraph Classic WSE-5400 (Atto).

### Viral growth kinetics

The viral growth curve assay was conducted as described previously [19]. Briefly, MA104 cells or MA104 cells expressing specific viral proteins (7.5 × 10⁴ cells/well) were seeded in 24-well plates and then infected for 1 h at 37°C at an MOI of 0.01. After washing three times with PBS, DMEM containing 0.5 μg/mL trypsin was added for the designated times. Cells were subjected to three freeze-thaw cycles, and viral titers were determined using the procedure described above.

### Virus passage in MA104 cells

MA104 cells (1.5 × 10⁵ cells/well) were plated in 12-well plates. After 24 h, the cells were infected with the single-round viruses at an MOI of 1.0. The cells were then washed with PBS, cultured in DMEM/0.5 μg/mL trypsin for 7 days, and then submitted to three freeze-thaw cycles. Then, 10% of the resulting lysate was transferred to fresh MA104 cells under the same conditions. This process was repeated for five sequential passages, with titers measured at each passage.

### Immunofluorescence assay

For confocal imaging, MA104 cells (7.5 × 10⁴ cells/well) were seeded onto coverslips in 24-well plates. The cells were infected with virus at an MOI of 1.0 and, at 8 h post-infection, were fixed and permeabilized as described above. Appropriate primary antibodies were applied, followed by fluorescence-labeled secondary antibodies. Nuclei were stained with Hoechst 33342. Images were acquired under a C2 + Eclipse Ti2 confocal microscope (Nikon).

### Detection of NLuc activity

MA104 cells (1 × 10⁴ cells/well) in 96-well plates were infected at an MOI of 1.0. After a 1 h incubation at 37°C, the cells were washed three times with PBS and cultured in DMEM supplemented with 5% FBS for the designated times. Cells were then freeze-thawed twice and NLuc activity measured in a NanoGlo Luciferase Assay (Promega).

### Virus challenge experiments

The virus challenge experiment using mice was conducted essentially as described previously [19]. To investigate viral replication *in vivo*, 3-week-old BALB/c mice were infected orally with 2 × 10⁶ FFU of virus. At 2 days post-infection, the cecum was collected, homogenized using a bead shocker, and subjected to viral titration. To evaluate pathogenicity, 4- or 5-day-old BALB/c pups were inoculated orally with 2 × 10⁵ FFU of virus. UV-inactivated virus was prepared by exposing samples to 1000 mJ/cm². Body weight and diarrhea symptoms were recorded daily. Diarrhea was defined as described previously [56]. For histopathological analysis, small intestinal sections were collected from mice at 2 days post-infection, fixed with 4% paraformaldehyde, and examined after HE staining. HE staining was outsourced to Genostaff.

### Immunization of mice

Neonatal (4- or 5-day-old) and 4-week-old BALB/c mice received three oral doses of virus at 3-week intervals. Two weeks after the second and/or third immunization, whole blood samples were harvested and serum isolated. The sera were subjected to enzyme-linked immunosorbent assay (ELISA) and neutralization testing as described below.

### ELISA

ELISA was conducted as described previously [19]. Briefly, CsCl gradient-purified, triple-layered rSA11 virions were coated onto 96-well MaxiSorp plates (Thermo Fisher Scientific) overnight at 4°C in carbonate buffer (15 mM Na₂CO₃, 7 mM NaHCO₃, pH 9.6). After washing with PBS/0.05% Tween (ELISA wash buffer), the plates were blocked with ELISA diluent (PBS/1% BSA/0.05% Tween) and then incubated for 1 h with sera from immunized mice (1:100), followed by HRP-conjugated anti-mouse IgG or IgA (Abcam) for 1 h at 37°C. Color development was achieved with 3,3′,5,5′-tetramethylbenzidine (Sigma-Aldrich), and absorbance was measured at 450 nm using a Cytation 5 Cell Imaging Multi-Mode Reader (Agilent).

### Neutralization assay

MA104 cells were plated in 96-well plates (1 × 10⁴ cells/well). On the following day, serially-diluted antibodies were mixed with 100 FFU of HiBiT-expressing rotavirus [51] and incubated at 37°C for 1 h, after which the mixtures were transferred onto cell monolayers. On the next day, cells were subjected to two freeze-thaw cycles, and HiBiT activity was measured using the Nano Glo HiBiT Lytic Detection System (Promega). Neutralization (%) was calculated based on HiBiT signal reduction, and neutralizing titers were expressed as the highest dilution yielding 50% inhibition.

### Passage of rSA11-VP4-defective in MA104-VP4

MA104-VP4 cells (1.5 × 10⁵ cells/well) were seeded in 12-well plates. After 24 h, cells were infected with rSA11-VP4-defective at an MOI of 0.01. Cultures were maintained for 7 days and then subjected to three freeze-thaw cycles. Subsequently, 1% of the supernatant was transferred to fresh MA104-VP4 cells in DMEM/0.5 μg/mL trypsin. This process was repeated 20 times, and the final viral stock was analyzed by next-generation sequencing (see below).

### Library preparation and sequencing

Viral RNA was denatured at 95°C for 90 s. cDNA synthesis was subsequently carried out using the GenNext RamDA-seq Single Cell Kit. The resulting cDNA was then utilized for library preparation with the Illumina Nextera XT DNA Library Preparation Kit, following the manufacturer’s protocol. Paired-end sequencing (101 bp) was performed on the Illumina NovaSeq 6000 platform.

### Genome assembly

Raw sequencing reads were processed by adapter trimming using Trimmomatic v0.38. The trimmed reads were then subjected to *de novo* assembly using CLC Genomics Workbench version 24.0.2, and the genome sequence was constructed.

### Statistical analysis

Data were analyzed using GraphPad Prism 9 (GraphPad Software, Inc.). Unless otherwise specified, all values represent means ± standard deviation from at least two independent experiments, each performed with three biological replicates. Statistical significance was defined as a P value of <0.05.

## Supporting information

S1 Fig Peak viral titers of rSA11-VP7-ΔDomain II and rSA11-VP7-Δ563-bp. Titers of the stock viruses at passage 2 are shown in the graph. Data are from a single experiment.
(DOCX)

S2 Fig Growth of rSA11-VP7-defective-P20 in MA104-VP7 cells. MA104-VP7 cells were infected with rSA11-VP7-defective-P2 or rSA11-VP7-defective-P20 at an MOI of 0.01 and harvested at the designated times. A t-test was used to evaluate significance, with P < 0.05 considered significant. ns = not significant; *P < 0.05.
(DOCX)
