# Supplementary material for: Single-round infectious rotaviruses with deletions of VP7 or VP4 genes, based on SA11 and WC3 strain backbones, and their potential use as viral vectors
Source: PLoS Pathog. 2025 Sep 15;21(9):e1013484. doi: 10.1371/journal.ppat.1013484 (PMC12435675; doi:10.1371/journal.ppat.1013484)
Supplement: S1 Fig — Titers of the stock viruses at passage 2 are shown in the graph. Data are from a single experiment. (DOCX) [file ppat.1013484.s001.docx]

**S1 Fig. Peak viral titers of rSA11-VP7-ΔDomain II and rSA11-VP7-Δ563-bp.**Viral titers of the stock viruses at passage 2 are shown in the graph. Data are from a single experiment.
