# Supplementary material for: Single-round infectious rotaviruses with deletions of VP7 or VP4 genes, based on SA11 and WC3 strain backbones, and their potential use as viral vectors
Source: PLoS Pathog. 2025 Sep 15;21(9):e1013484. doi: 10.1371/journal.ppat.1013484 (PMC12435675; doi:10.1371/journal.ppat.1013484)
Supplement: S2 Fig — MA104-VP7 cells were infected with rSA11-VP7-defective-P2 or rSA11-VP7-defective-P20 at an MOI of 0.01 and harvested at the designated times. A t-test was used to evaluate significance, with P < 0.05 considered significant. ns = not significant; *P < 0.05. (DOCX) [file ppat.1013484.s002.docx]

**S2 Fig. Growth of rSA11-VP7-defective-P20 in MA104-VP7 cells.**

MA104-VP7 cells were infected with rSA11-VP7-defective-P2 or rSA11-VP7-defective-P20 at an MOI of 0.01 and harvested at the designated times. A t-test was used to evaluate significance, with P < 0.05 considered significant. ns = not significant; *P < 0.05.
